# Augmentation-Mastopexy: Analysis of 95 Consecutive Patients and Critical Appraisal of the Procedure

**DOI:** 10.3390/jcm12093213

**Published:** 2023-04-29

**Authors:** Isabel Zucal, Mathias Tremp, Dominik Duscher, Raphael Wenny, Maximilian Zaussinger, Alexander Kutz, Andrea Pagani, Georg M. Huemer

**Affiliations:** 1 Department of General Surgery, Cantonal Hospital Aarau, 5001 Aarau, Switzerland; 2 Department of Plastic, Reconstructive, Aesthetic and Hand Surgery, University Hospital Basel, 4031 Basel, Switzerland; 3 TF Plastic Surgery and Longevity Center, Herzogstrasse 67, 80803 Munich, Germany; 4 TF Plastic Surgery and Longevity Center, Dorotheergasse 12, 1010 Vienna, Austria; 5 Department of Plastic, Reconstructive, Hand and Burn Surgery, BG-Trauma Center, Eberhard Karls University Tübingen, 72076 Tübingen, Germany; 6 Section of Plastic, Aesthetic and Reconstructive Surgery, Medcampus III, Kepler University Hospital, 4020 Linz, Austria; 7 Medical University Department, Division of General Internal and Emergency Medicine Cantonal Hospital Aarau, 5001 Aarau, Switzerland; 8 Division of Pharmacoepidemiology and Pharmacoeconomics, Department of Medicine, Brigham and Women’s Hospital and Harvard Medical School, Boston, MA 02114, USA; 9 Department of Orthopedics, Traumatology and Hand Surgery, Hospital of Bolzano—SABES, 39100 Bolzano, Italy

**Keywords:** aesthetics, breast, breast implants, mammaplasty, plastic and reconstructive surgery

## Abstract

Single-stage mastopexy-augmentation has been demonstrated to be a safe procedure. However, revisions may still be necessary. We evaluate 95 consecutive patients undergoing mastopexy-augmentation and introduce a new surgical technique for the procedure: the modified dual plane technique. In this retrospective study, 95 patients (mean age 34 ± 11 years) underwent mastopexy-augmentation between 2009 and 2019. The procedures were classified as subglandular, dual plane, or modified dual plane technique. The outcome measures included major and minor complications. A total of 19 patients underwent a subglandular procedure, 32 patients a dual plane procedure, and 44 patients a modified dual plane procedure. We observed a high overall complication rate in the subglandular group (n = 12, 63%), dual plane group (n = 15, 47%), and modified dual plane group (n = 10, 23%). Complications leading to implant loss/change occurred in seven patients in the subglandular group (37%), six patients in the dual plane group (19%), and no patient in the modified dual plane group. While we observed a high complication rate in patients undergoing mastopexy-augmentations, the modified dual plane technique was associated with a lower complication rate.

## 1. Introduction

Many patients desiring breast augmentation require mastopexy, too. Vice versa, many patients desiring mastopexy also want an additional breast augmentation. Especially post-bariatric patients suffering from severe ptosis and breast volume reduction profit from combined mastopexy-augmentation [1]. In fact, one main advantage of combined mastopexy-augmentation is that patients need to undergo only one surgical intervention, and an immediate result is achieved. On the other hand, there have been concerns about simultaneous mastopexy-augmentations and implant placement because of the relatively high complication rate including severe wound healing problems, hematoma, double bubble deformity, implant malposition, and eventually implant loss, especially in massive weight loss patients [2,3]. For these patients, various techniques of combined mastopexy-augmentation have been described, with varying incisional patterns (periareolar, vertical, or inverted T incisional approach) and varying implant placement plane (subglandular, dual plane, submuscular) or autologous tissue augmentation with or without autologous fat grafting [4,5,6,7].

In this study, we analyzed 95 consecutive cases that underwent mastopexy-augmentations between November 2009 and July 2019 with the three surgical techniques (subglandular, dual plane, and modified dual plane technique), and we evaluated safety and complications especially for the aforementioned modified dual plane implant placement.

## 2. Materials and Methods

This was a retrospective study of 95 consecutive female patients (mean age 34 ± 11 years, range 18–60 years) who underwent mastopexy-augmentation between November 2009 and July 2019. Eighty-nine of the interventions were carried out at the Section of Plastic, Aesthetic, and Reconstructive Surgery at the Kepler University Hospital, Johannes Kepler University in Linz, Austria, by the senior doctor and his assistant doctors. Six further interventions were performed at the Department of Plastic, Reconstructive, Aesthetic, and Hand Surgery at the University Hospital of Basel, Switzerland. Written and informed consent was collected from all patients, and the study was approved by the local review board of both institutions. Patients undergoing combined mastopexy-augmentation with subglandular, dual plane, or modified dual plane implant placement were included. Patients actively smoking and/or patients with current signs of infection were excluded. Specifically, only patients who quit smoking at least 3 weeks before the surgery were included. The study population was divided in three groups on the basis of the three aforementioned surgical approaches: group I = subglandular implant placement, group II = dual plane approach [8], and group III = modified dual plane implant placement [9]. Because of the retrospective design of the study, no deliberate group allocation was performed, and all patients who had undergone one of the aforementioned procedures were included. 

### 2.1. Surgical Technique Modified Dual Plane

**Figure 1:**
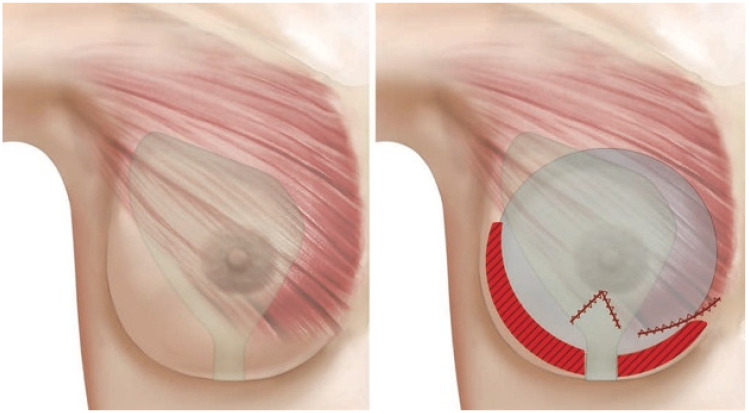
The Modified dual plane dissection (From Huemer GM, 2018). On the left side, the shape of the subglandular pocket is represented. On the right, incisions of the pectoralis major muscle are shown as zig-zag red lines. Releasing the sternal insertion of the muscle, it stays attached to the gland and does not slide cranially when placing the implant. Incising the middle part of the pectoralis muscle, breast animation deformity is prevented. Finally, the preservation of the inframammary fold is crucial and this region is marked in red. Reprinted with permission from Huemer et al. [9]. 2018, Wolters Kluwer Health, Inc.

The modified dual plane approach was first described by Huemer et al. in 2018 for Motiva implants. First, a triangular pocket with a cranial basis is built under the mammary gland; as a result, the gland remains attached only in the inferior quadrants. Then submuscular dissection is started. This is performed similarly to the dual plane technique except for the release of the sternal insertion, which is performed only in the modified dual plane approach. Because of the modified subglandular dissection, the free sternal muscle insertion is still attached to the gland. This allows for both sufficient sliding of the gland and a more stable positioning of the pectoralis major muscle, preventing it from sliding cranially (window-shading) during the implant placement. To prevent breast animation deformity, the inferior border (abdominal insertion) of the pectoralis major muscle is incised in the central part. Fixation of the inframammary fold is recommended [9]. This new approach is represented in Figure 1. A video of the procedure is provided in the Supplementary Materials as well (Video S1).

### 2.2. Study Outcomes

We analyzed the patients’ demographics including age, preexistent risk factors, weight, height, and body mass index (BMI) at the time of surgery. Surgery-specific data such as incision technique (periareolar, circumvertical, or inverted T); implant placement (subglandular, dual plane, or modified dual plane); implant size for the right and left side, respectively; implant company; antibiotic intake; number of drains; and intraoperative time were assessed. With regard to drains, no drains were used in group III because over time there was increased evidence in the literature showing no advantage of drains in breast surgery [10,11,12,13]. Moreover, we analyzed the outcome measures in terms of complications such as rupture, capsular contracture, dislocation, double bubble deformity, hematoma, seroma, wound dehiscence, wound infection, and intense scarring, as well as revisions, postoperative visits, and days of hospitalization. In particular, we observed if complications led to implant loss or not.

### 2.3. Data Analysis

We descriptively examined the patient characteristics among all the intervention groups using a one-way ANOVA test for comparison of demographic data. The significance level was set to *p* ≤ 0.05. The study outcomes, i.e., complications, were studied fitting a multivariable regression model adjusting for BMI, implant size, surgeon, and intraoperative time. We assessed odds ratios (OR) between the modified dual plane method (group III) and the subglandular method (group I), or between the modified dual plane method (group III) and the dual plane method (group II). To address a potential bias of repeated testing effects, we adjusted for multiple comparisons using the Bonferroni method. For outcomes with zero events in one of the groups, unadjusted ORs were calculated with the Haldane-Anscombe method to cope with the absence of cases. Statistical significance was based on 95% confidence intervals (CI), and all *p*-values were two-sided. All the statistical analyses were performed using Microsoft^®^ Excel (2019) or Stata, version 17.0 (StataCorp LLC).

## 3. Results

In total, we identified 19 patients with a subglandular implant placement (group I), 32 patients with a dual plane placement (group II), and 44 patients with a modified dual plane implant placement (group III). Detailed group classification can be viewed in Table 1.

### 3.1. Demographics

All the patients were female and had a mean age of 33.6 ± 11.2 years (range: 18–60 years) at the date of surgery. The mean weight at the date of surgery was 66.6 ± 10.1 kg, and the BMI was 23.7 ± 4.4 kg/m^2^. In group I, the patients had a mean age of 33.0 ± 12.2 years at the date of surgery, and they had a mean weight of 72.2 ± 11.8 kg and mean BMI of 26.2 ± 4.2 kg/m^2^. Group II had a mean age of 32.8 ± 9.2 years, a mean weight of 64.3 ± 9.4 kg, and a mean BMI of 23.0 ± 3.0 kg/m^2^. Finally, group III had a mean age of 36.1 ± 9.4 years, a mean weight of 64.5 ± 5.5 kg, and mean BMI of 23.5 ± 1.9 kg/m^2^. Significant differences in the patients’ BMI and weight were observed, with patients in group I having a higher BMI and body weight (*p* < 0.01 for both) compared to group II and III, whereas there was no difference in age among the patient groups (*p* = 0.29). Detailed insight into these data is provided in Table 2.

**Figure 2:**
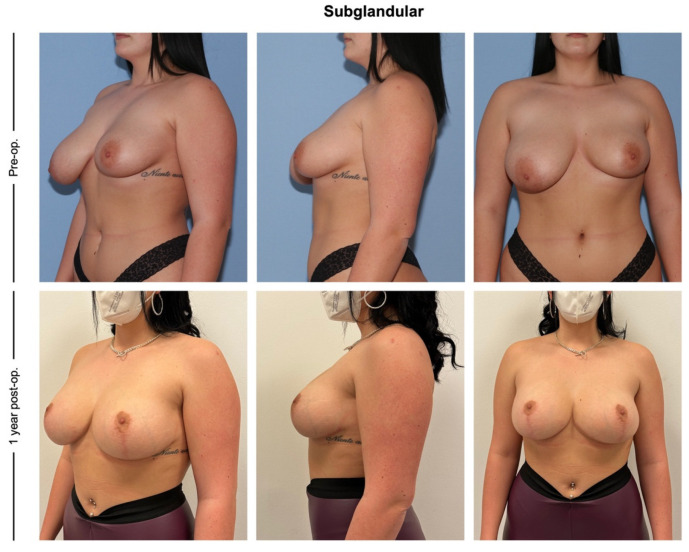
The subglandular technique (group I). In the pre-operative pictures, a breast asymmetry can be observed (right > left). Together with the patient, a subglandular approach was chosen. Post-operative pictures 1 year after mastopexy-augmentation are presented. Symmetry and stable results were achieved.

**Figure 3:**
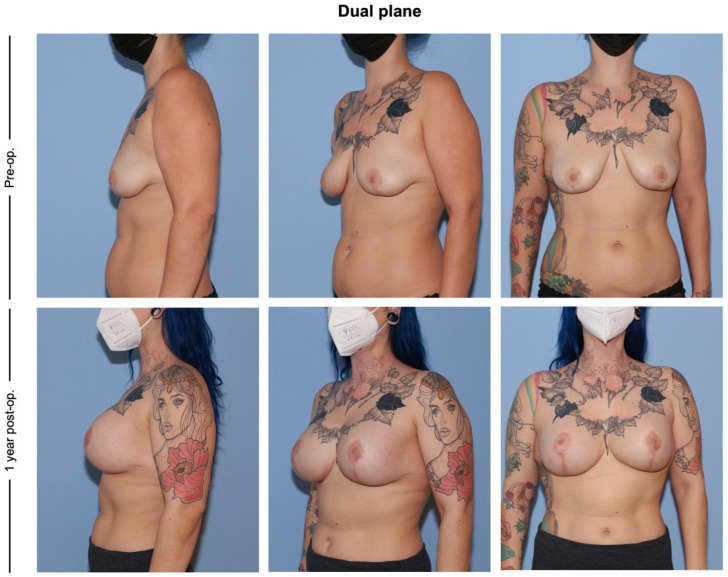
The dual plane technique (group II). This patient previously had a mastopexy and presented with bilateral ptosis and volume loss. A dual plane technique was performed. The postoperative pictures show a satisfying result with no recurrence of ptosis.

**Figure 4:**
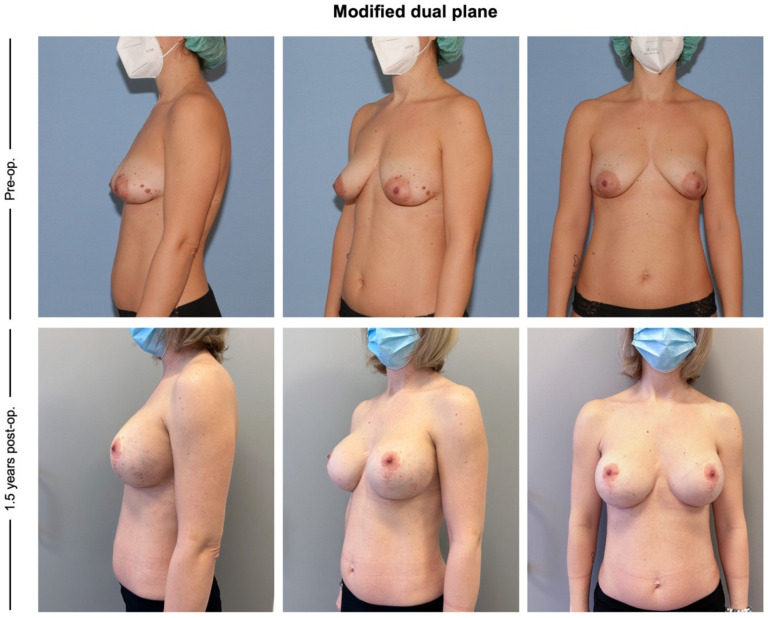
The modified dual plane technique (group III). Pre-operative pictures demonstrate bilateral ptosis. Postoperative pictures 1.5 years after modified dual plane mastopexy-augmentation show symmetrical breasts and no signs of recurrent ptosis.

Pre-and postoperative pictures of each group are presented in Figure 2, Figure 3 and Figure 4.

**Figure 5:**
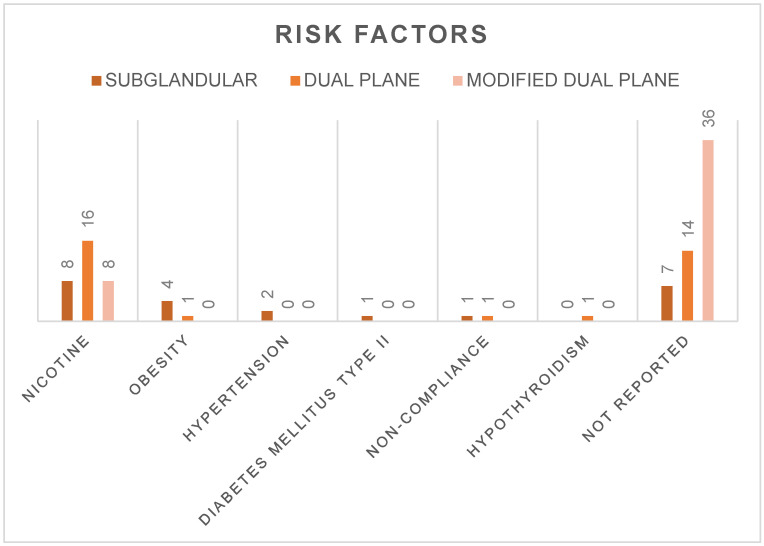
Risk factors. Risk factors such as history of tobacco use, obesity, hypertension, diabetes mellitus type II, non-compliance, hypothyroidism, and not reported are shown for each study group (I–III: subglandular, dual plane, modified dual plane), respectively.

Regarding risk factors, a total of 32 patients (33.7%) had a history of smoking: eight patients in group I, sixteen in group II, and eight in group III. Moreover, five patients (5.3%) were obese: four patients in group I and one patient in group II, respectively. No patient in group III was obese. Two patients (2.1%) suffered from hypertension, and they were both in group I. Also, diabetes mellitus type II was present in one patient (1.1%) from group I. Non-compliance was considered a risk factor as well, and it was registered in two patients (2.1%): one patient in group I and one patient in group II, respectively. Finally, one patient in group II (1.1%) suffered from hypothyroidism. Unfortunately, 57 patients (60.0%) presented an unknown history of risk factors, so we lack these data. A graphic illustration of the risk factors is provided in Figure 5.

### 3.2. Surgical Approaches and Hospitalization

Regarding surgical techniques, the incisional pattern was classified into periareolar, circumvertical, and inverted T technique. Six patients (18.8% of the group) in group II had a periareolar incision, whereas no patient in group I or III had this. Most of the patients had a circumvertical incision: six patients (31.6%) in group I, sixteen patients in group II (50.0%), and 36 patients (81.8%) in group III. The inverted T approach was common as well: thirteen patients (68.4%) in group I, ten patients (31.1%) in group II, and eight patients (18.2%) in group III. These data are represented in Table 3.

The mean implant size was 326.6 ± 92.8 cc in group I, 314.1 ± 61.2 cc in group II, and 367.7 ± 68.4 cc in group III. Regarding the implant type, three Polytech^®^ implants were used in group I, five in group II, and nine in group III. Mentor^®^ implants were used for eleven patients in group I, twelve in group II, and five in group III, whereas Eurosilicone^®^ implants were used in five cases in group I, fifteen in group II, and none in group III. Motiva^®^ implants were applied in 18 patients in group III. Also, in group III, Allergan^®^ was used in four patients, Sebbin^®^ implants in seven patients, and for one patient data were missing. Round implants were used in 14 patients in group I (73.7%), 17 patients in group II (53.2%), and 36 patients in group III (81.8%). A detailed overview is provided in Table 4.

Prophylactic antibiotics were given to all (100.0%) patients in all groups. The mean intraoperative time was 02:33 ± 00:59 h in group I, 02:08 ± 00:55 h in group II, and 01:54 ± 00:57 h in group III. The mean stay of the drains was 1.2 ± 1.5 days in group I and 0.5 ± 1.1 days in group II; group III did not have any drains. The patients in group I had a mean hospitalization stay of 4.1 ± 1.9 days and 5.0 ± 7.1 postoperative visits, and the patients in group II had a mean hospitalization stay of 4.0 ± 1.7 days with on average 3.0 ± 1.4 postoperative visits. Finally, group III had a mean hospitalization stay of 1.9 ± 1.8 days with 2.5 ± 1.2 postoperative visits. The shorter hospitalization time for group III is partly explained by fewer complications and the fact that drains were not used. Since no patients in group I and II were discharged with drains, this led to a longer hospitalization time. Considering all the groups, the mean follow-up was 32 months (range 14–72 months). 

### 3.3. Complications

Complications occurred for a total of 12 patients in group I (63.2%), 15 patients in group II (46.9%), and 10 patients in group III (22.7%). Surgical revision was required in eight patients in group I (42.1%), ten patients in group II (31.3%), and two patients in group III (4.5%). The complications led to implant loss/change in seven patients in group I (36.8%) and six patients in group II (18.8%). No patient in group III had a complication with implant loss or change. In group III, there were significantly fewer complications compared to group I (OR 0.09, CI 0.01–0.90), and compared to group II, there were numerically fewer complications, but the difference was not significant (OR 0.15, CI 0.02–1.25). Accordingly, significantly fewer revisions were required in group III compared to group I (OR 0.05, CI 0.01–0.42) and II (OR 0.09, CI 0.01–0.72). Moreover, fewer complications leading to implant loss/change were observed in group III compared to group I (OR 0.02, CI 0.00–0.35) and group II (OR 0.05, CI 0.00–0.85). An overview is provided in Table 5.

Specifically, wound dehiscence was the most common complication in all the groups. It occurred in eight patients in group I (42.1%), five patients in group II (15.6%), and five patients in group III (11.4%). The median time to occurrence was 14 (7–28) days. In group III, numerically fewer cases of wound dehiscence were observed compared to group I (OR 0.05, CI 0.00–1.15) and group II (OR 0.29, CI 0.02–4.98), respectively. With regard to wound infection, this occurred in five patients in group I (26.3%), three patients in group II (9.4%), and two patients in group III (4.5%). The median time to occurrence was 14 (7–21) days. Because of the small sample sizes and the high impact of confounding factors, no significant differences were found comparing the groups with regard to specific complications including hematoma (median time to occurrence < 5 h), seroma (median time to occurrence 17.5 (14–21) days), capsular contracture (median time to occurrence 40 (36–48) months), double bubble deformity (median time to occurrence 39 (36–45) months), intense scarring (median time to occurrence 6 (0.5–36) months), and rupture (time to occurrence 40 months). A detailed description of the frequencies of complications and odds ratios (CI) is given in Table 6.

## 4. Discussion

Various techniques of mastopexy-augmentation are described in the literature. On the one hand, autologous mastopexy-augmentation techniques are promoted in the literature because of their safety and natural aesthetic results [3]. For instance, a dermal or glandular flap can be created to avoid pseudoptosis in the long term and to provide breast support [14]. Di Summa et al. described a technique based on the creation of dermal triangular flaps in order to create lower pole fullness and prevent ptosis [15]. Instead of an implant placement, autologous fat grafting can be used as well. Gentile et al. described natural and aesthetically pleasing results even in the correction of breast deformities with or without adipose-derived stem cell enrichment [4,7].

In this study, we focused on patients desiring a substantial breast augmentation along with mastopexy and we compared three different planes of implant placement:

In the subglandular approach, the breast implant is placed over the pectoralis major muscle and under the mammary gland. This technique is suitable for patients with sufficient upper pole fullness [16]. One of the main advantages of this technique is that there are lower rates of implant displacement and animation deformity compared to submuscular or dual plane approaches [17].

In the dual plane approach, the breast implant is partially placed under the pectoralis major muscle. In this case, the abdominal insertion of the pectoralis major muscle is dissected along the inframammary fold, preserving the sternal insertion, and the implant is placed underneath the muscle without closing the muscle and suturing it back to its abdominal insertion. Three types of dual plane are commonly described: In type I dual plane, no additional subglandular dissection is performed. In type II dual plane, the mammary gland is dissected and detached from the pectoralis major muscle up to the lower border of the areola. Finally, in type III dual plane, the subglandular dissection reaches the upper border of the areola. The dual plane technique is claimed to provide a more natural result, especially in skinny patients, in whom the upper pole fullness is missing [8]. Moreover, the technique is claimed to reduce capsular contracture and hematoma compared to the subglandular implant placement [17,18,19].

In the modified dual plane, as described in the Materials and Methods section, the caudal part of the sternal insertion of the pectoralis major muscle is released and a triangular incision is performed at the abdominal insertion [9].

In this study, we found a higher complication rate (*p* = 0.01) in group I with the subglandular implant placement (63.2%), followed by group II with the dual plane technique (46.9%); the lowest complication rate was registered in group III with the modified dual plane approach (22.7%). Wound dehiscence was the most common complication affecting 42.1% of the patients in group I, 15.6% in group II, and 11.4% in group III. Infection occurred in 26.3% of the patients in group I, 9.4% in group II, and 4.5% in group III. There was a numerically higher wound dehiscence and infection rate in group I.

Moreover, in the group with modified dual plane, we noticed that it not only had the lowest complication rate, but bigger implants were used (367.7 ± 68.4 cc) compared to groups I and II (326.6 ± 92.8 cc and 314.1 ± 61.2 cc respectively) with the subglandular and dual plane approaches. In this regard, some authors recommend smaller implants for lower complication rates [20,21], but other studies did not find a correlation between the size of the implant and the complication rate [22,23,24].

Finally, the modified dual plane technique was the fastest surgical approach with a mean intraoperative time of 01:54 ± 00:57 h compared to subglandular and dual plane surgery (02:33 ± 00:59 h and 02:08 ± 00:55 h respectively). Reduced intraoperative time is positively correlated with a better patient outcome [25,26] and is a cost-saving factor.

In a review of 615 consecutive patients who underwent single-stage mastopexy-augmentation between 1992 and 2011, Stevens et al. concluded that combined surgery can be safe and effective with appropriate patient selection and experienced surgeons. He reported a revision rate of 16.9% with poor scarring (5.7%), wound-healing problems (2.9%), and deflation of saline implants (2.4%) being the most common complications [2]. Accordingly, Qureshi et al. recommended mastopexy-augmentation as a safe option for correction of shape and volume [5]. A review by Khavanin et al. including 23 studies and 4856 cases reported a pooled total complication rate of 13.1%; recurrent ptosis was the most common complication (5.2%), followed by poor scarring (3.7%). Infection, hematoma, and seroma had incidences of less than 2% each. The revision rate was 10.7% [27]. In a study of 384 patients, Cárdenas-Camarena et al. reported a complication rate of 18%, and appropriate patient and technique selection were emphasized [28]. Swanson reported a complication rate of 32.9% for vertical augmentation-mastopexy with a revision rate of 15.5%. Complications included persistent ptosis, delayed wound healing, scar deformities, and asymmetry. In this survey, 13.3% of the patients had persistent nipple numbness. Of the 252 analyzed patients, 90 participated in a survey and 94.4% reported that they would repeat surgery. In conclusion, the author confirmed the safety and applicability of this technique [29]. In summary, variable complication rates are reported in the literature, but all the authors consider mastopexy-augmentation a safe procedure.

With regard to the outcome of revisions in mastopexy-augmentation, Spear et al. published a study of 20 patients who underwent revision surgery. Of the 34 operated breasts, ten implants were originally subglandular and 24 partially or totally submuscular. Capsular contracture in 11 patients (55%), nipple ptosis in 11 (55%), implant malposition in seven (35%), dissatisfaction with implant size in six (30%), poor scarring in five (25%), breast ptosis in 4 (20%), nipple malposition in two (10%), and patient preference in one (5%) were reasons for revision. Five of the 20 patients (25%) had a revision of a previous revision [19]. In our study, similarly high complication rates were found. However, when comparing the complication rates of different studies, consideration of the definition of complications, inclusion of long-term complications, inclusion of massive weight loss patients, smoking status, etc. is of utmost importance. Moreover, as most patients were operated upon by the senior author and developer of the modified dual plane technique, fewer complication rates and better outcomes were expected with this technique compared to the others.

A particular patient group to consider in mastopexy-augmentation is the massive weight loss patient population. In this regard, Coombs et al. reported an implant malposition rate of 61.9% within 12 months postoperatively and a revision rate of 6.6%. Implant malposition correlated significantly to higher current body mass index but not to implant size, according to this study [30].

With regard to the limitations of our study, the patients in group I had a significantly higher BMI and weight compared to those in group II and group III. It is known that elevated BMI and body weight represent risk factors for higher complication rates in surgery in general [31,32,33,34] and especially in breast reconstruction [34]. Hence, this may contribute to the higher number of complications we encountered in group I. The effect of overweight and obesity on postoperative complications has been widely studied in the past few decades. In fact, it has been shown that obese patients have a reduced adipose tissue blood flow [35,36,37] leading to chronic hypoxia of tissue and thus to tissue dysfunction and inflammation [38,39,40]. These studies report that oxygen supply is reduced in subcutaneous fat, probably causing delays in wound healing and increased infection risk after surgery. Moreover, obese and especially post-bariatric patients additionally tend to be malnourished [41,42]. Deficiencies in proteins and micronutrients such as vitamins and minerals contribute to impaired wound healing, as adequate collagen synthesis and granulation tissue formation are not supported [43,44]. Generally, simultaneous mastopexy-augmentations are common in post-bariatric patients, and for the aforementioned reasons patients have a higher risk for complications compared to healthy individuals. Thus, a high complication rate in surgical post-bariatric ptotic breast reconstruction is expected. In our study, we did not take into account whether a patient was post-bariatric or not, so this is surely an important limitation. However, in our study there were few post-bariatric patients, and most of the patients were of normal BMI and weight or slightly overweight (23.0 ± 3.0 kg/m^2^ and 64.3 ± 9.4 kg in group II, 23.5 ± 1.9 kg/m^2^ and 64.5 ± 5.5 kg in group III, 26.2 ± 4.2 kg/m^2^ and 72.2 ± 11.8 kg in group I).

Not including active smokers is another important limitation to the study, as this may have influenced the complications and outcomes substantially. However, patients who quit smoking only 3 weeks before surgery were considered non-smokers, and it was not recorded whether and when they started smoking again. Moreover, 57 (60%) of the patients had an unknown history of risk factors, which is a major limitation to the study.

Another limitation of the study was that we did not include subfascial and submuscular implant placement as a surgical technique, so we cannot exclude that those techniques may have lower complication rates compared to the modified dual plane technique. However, recent studies suggest a significantly increased risk of rupture when performing a totally submuscular plane approach [45]. In our study, we observed one rupture in the dual plane group (group II). Further limitations are the retrospective character of the study and the small patient number per study group. This substantially limited the comparison between groups. With only 19 patients in group I, we had a small patient cohort for the subglandular plane, and future studies with larger patient cohorts are required for the comparison and validation of a new technique. Moreover, we did not adjust for multiple testing. As a final limitation, the incisional approach (periareolar, circumvertical, and inverted T) was not evaluated separately for complications, but it is known that wound healing problems are especially encountered where incisions collide (which is the case with inverted T incision) as the wound edges are poorly vascularized [46].

The high complication rate in the subglandular approach may be explained by the unpredictable change in soft tissue when simultaneous mastopexy occurs. If the implant is placed in a submuscular or partially submuscular plane, it has better stability, and changes in the soft tissue above do not influence the implant; thus, deformities and wound dehiscence are prevented. In summary, in this study, a high complication rate was observed compared to other studies in the literature with regard to the subglandular and dual plane approaches. However, complications also depend on the surgeons’ experience with and adherence to the particular technique. The modified dual plane approach had a comparable outcome to other studies that approve single-stage mastopexy-augmentation as a safe and applicable technique. This means that our newly proposed modified dual plane technique is a valid option for patients who desire breast lift and increase in volume.

## 5. Conclusions

We observed a high complication rate in patients undergoing mastopexy-augmentations, especially in those undergoing the subglandular or dual plane approaches (63.2% and 46.9% respectively), whereas with the modified dual plane technique the complication rate was significantly lower (22.7%). We conclude that a new surgical technique resulting from the refinement of pre-existing augmentation-mastopexy techniques may be safe and even associated with fewer complications if the surgeon is familiar with them.

## Figures and Tables

**Table 1:** Group classification depending on the method of implant placement.

Group (I–III):	I (Subglandular)	II (Dual Plane)	III (Modified Dual Plane)
No. (%)	19 (20)	32 (34)	44 (46)

**Table 2:** Patients’ demographics including age, BMI, and weight at the date of surgery.

Patient Data (Mean ± SD)	Group I	Group II	Group III	*p*-Value
Age	33.0 ± 12.2	32.8 ± 9.2	36.1 ± 9.4	0.29
BMI	26.2 ± 4.2	23.0 ± 3.0	23.5 ± 1.9	<0.01
Weight	72.2 ± 11.8	64.3 ± 9.4	64.5 ± 5.5	<0.01

**Table 3:** Data about the incisional approach are represented for each group.

Incisional Techniques, No. (%)	Group I	Group II	Group III
Periareolar	0 (0.0)	6 (18.8)	0 (0.0)
Circumvertical	6 (31.6)	16 (50.0)	36 (81.8)
Inverted T	13 (68.4)	10 (31.3)	8 (18.2)

**Table 4:** Implant company overview for each group.

Implant Type, No. (%)	Group I	Group II	Group III
Polytech^®^	3 (15.8)	5 (15.6)	9 (20.5)
Mentor^®^	11 (57.9)	12 (37.5)	5 (11.4)
Eurosilicone^®^	5 (26.3)	15 (46.9)	0 (0.0)
Motiva^®^	0 (0.0)	0 (0.0)	18 (40.9)
Allergan^®^	0 (0.0)	0 (0.0)	4 (9.1)
Sebbin^®^	0 (0.0)	0 (0.0)	7 (15.9)
Unknown	0 (0.0)	0 (0.0)	1 (2.3)
Total	19	32	44

**Table 5:** Complication rates with and without implant loss and revision rates provided for each study group.

Complications, No. (%):	Group I	Group II	Group III	OR_III vs. I_ (95% CI)	OR_III vs. II_ (95% CI)
Patients with complications	12 (63.2)	15 (46.9)	10 (22.7)	0.09 (0.01–0.90)	0.15 (0.02–1.25)
Revisions	8 (42.1)	10 (31.3)	2 (4.5)	0.05 (0.01–0.42)	0.09 (0.01–0.72)
Implant loss/change	7 (36.8)	6 (18.8)	0 (0.0)	0.02 (0.00–0.35) *	0.05 (0.00–0.85) *

* corrected OR using Haldane-Anscombe ½ correction.

**Table 6:** Number of patients with complications and complication frequencies listed and compared.

Complication, No. (%)	Group I	Group II	Group III	OR_III vs. I_ (95% CI)	OR_III vs. II_ (95% CI)
Wound dehiscence	8 (42.1)	5 (15.6)	5 (11.4)	0.05 (0.00–1.15)	0.29 (0.02–4.98)
Wound infection	5 (26.3)	3 (9.4)	2 (4.5)	0.33 (0.03–3.54)	1.31 (0.12–14.90)
Hematoma	2 (10.5)	1 (3.1)	1 (2.3)	0.23 (0.01–4.91)	0.98 (0.03–33.43)
Seroma	1 (5.3)	0 (0.0)	1 (2.3)	0.26 (0.00–511.43)	2.24 (0.09–∞) *
Capsular contracture	1 (5.3)	3 (9.4)	0 (0.0)	0.14 (0.00–3.56) *	0.09 (0.00–1.90) *
Double bubble deformity	0 (0.0)	2 (6.3)	2 (4.5)	2.29 (0.11–∞) *	0.47 (0.03–6.91)
Dislocation	2 (10.5)	3 (9.4)	1 (2.3)	0.21 (0.01–4.46)	0.27 (0.01–4.89)
Intense scarring	2 (10.5)	3 (9.4)	0 (0.0)	0.08 (0.00–1.72) *	0.09 (0–1.90) *
Rupture	0 (0.0)	1 (3.1)	0 (0.0)	0.44 (0–∞) *	0.24 (0.00–5.98)

* corrected OR using Haldane-Anscombe ½ correction.

## Data Availability

The data presented in this study are available on request from the corresponding author. The data are not publicly available due to privacy.

## Supplementary Materials

Video S1: The modified dual plane technique. https://zenodo.org/record/7758017#.ZEzLKC221R0.
